# Inquiry-Team-Based Lab Course Design Enhances Underrepresented Undergraduate Predictors of Persistence in the Sciences

**DOI:** 10.1007/s40670-024-02014-y

**Published:** 2024-03-01

**Authors:** Nicholas L. Denton, Amy E. Kulesza

**Affiliations:** 1 Division of Pharmacy Education and Innovation, The Ohio State University College of Pharmacy, Columbus, OH USA; 2 Center for Life Sciences Education, The Ohio State University, Columbus, OH USA

**Keywords:** Inquiry-based learning, Team-based learning, Laboratory skills, Professional identity formation, Diversity equity and inclusion

## Abstract

**Introduction:**

Persistence in Science, Technology, Engineering, and Mathematics (STEM) may be promoted in underrepresented student populations by implementing an authentic inquiry-team-based learning (ITBL) STEM laboratory course design.

**Methods:**

Between Spring 2021 and Spring 2022, the research team compared junior and senior undergraduates enrolled in an ITBL-based pharmaceutical science lab course to a comparative student population enrolled in a traditionally designed biology lab course. At the end of either STEM lab course, students completed the experimentally validated Persistence in the Sciences (PITS) survey and an open-ended question asking them to recount a moment that validated or questioned their science identity determined the effect of the ITBL STEM lab course design on factors that may impact underrepresented students’ indicators of science identity formation and persistence in STEM.

**Results:**

Students taking an ITBL-based pharmaceutical sciences lab course demonstrated higher scores on the persistence in the sciences instrument compared to students in the traditionally designed biology lab. Interestingly, different underrepresented student communities scored differently among the six factors. Multiple mechanisms of validating science identity were cited by students such as through gaining confidence in individualistic laboratory performance, collaborating through learning barriers, and fostering confidence and societal impact in a future career in pharmacy.

**Conclusion:**

The pharmaceutical sciences ITBL lab offered a collaborative, growth-promoting environment with experiments that are authentic to perspective pharmacists, which resulted in students reporting higher persistence in the sciences scores indicative of feeling like a pharmacist such as project ownership content/emotion, science identity, and networking across various student demographics.

## Introduction

Undergraduates in medical science programs must first build their identity as scientists before ever applying for the post-bachelor programs that will help them enter their career of choice. To build that identity, students must learn to think, feel, and act like science professionals. This occurs when students are socialized to the field’s values and behaviors through the curriculum, interactions with peers and instructors, and reflection [1, 2]. A strong professional identity has been linked with future career success and other affective benefits, such as mental well-being and social connections [3]. Moreover, this developed sense of science identity along with a feeling of community in Science, Technology, Engineering, and Mathematics (STEM) is a strong predictor for student persistence in the sciences, especially when challenged with rigorous medical science programs at all levels [4].

For pre-pharmacy students, undergraduate STEM lab courses are often the influential experiences that shape their perspective pharmacist professional identity because of the perceived authenticity of lab experiences and socialization with both fellow perspective pharmacists and practicing instructors. Lab courses have goals that strongly align with pharmacist behaviors such as gaining pharmaceutical science knowledge, proficiency in laboratory skills, overcoming challenges, developing curiosity, and making meaningful contributions to a research team. Pharmacist values such as ownership in their practice, intrinsic motivation toward pharmacy, commitment to continuous learning, pharmacist self-identity, sense of pharmacy community, and networking are also strong predictors for student persistence in the sciences [1, 2, 4].

However, students from minoritized populations face additional challenges of identity threat that undermine student achievement in persistence-fostering goals. Traditional team-based laboratory courses that assess more heavily on first-time lab performance metrics rather than assess inquiry-based data interpretation do not provide the psychological safety for students to take risks and learn from mistakes. Similarly, inquiry-based courses that lack team-based explicit reflection on the authenticity of the laboratory experience to the behaviors and values of the discipline will disproportionately underserve students that start out with more self-doubt in their lab abilities and fewer positive socializations with pharmacists than their peers [5]. For example, minoritized students that concede work to a less identity-threatened lab partner during high-stake labs may compromise their sense of project ownership to avoid a poor grade and being perceived as incompetent. This may also result in minoritized students struggling through assignments alone rather than collaborating with peers or instructors, resulting in frustration in lab, exacerbated self-doubt, and dislike in practicing the scientific process [6, 7]. Opportunity gaps in first-generation students’ prior lab experiences and lack of exposure to the “hidden curriculum” of STEM education such as undergraduate research, office hours, and extension policies create a disconnect in student-instructor expectations that further challenges minoritized student persistence in pharmacy, resulting in only < 65% of women and < 60% of black students that initially enroll in an undergraduate STEM major earning a STEM degree [8]. This loss of promising scientists in STEM education has resulted in an immediate loss of enrollment in medical science programs, but also a greater loss in medical discoveries and innovations, missed economic opportunities, and exacerbated healthcare disparities. Because this exclusion of minoritized students has been intentionally rooted in the educational system, course design moving forward must actively work to change these practices and use approaches that support minoritized students, increase a sense of belonging, and foster science identity in students to promote persistence in STEM [9].

## Objective

**Fig. 1:**
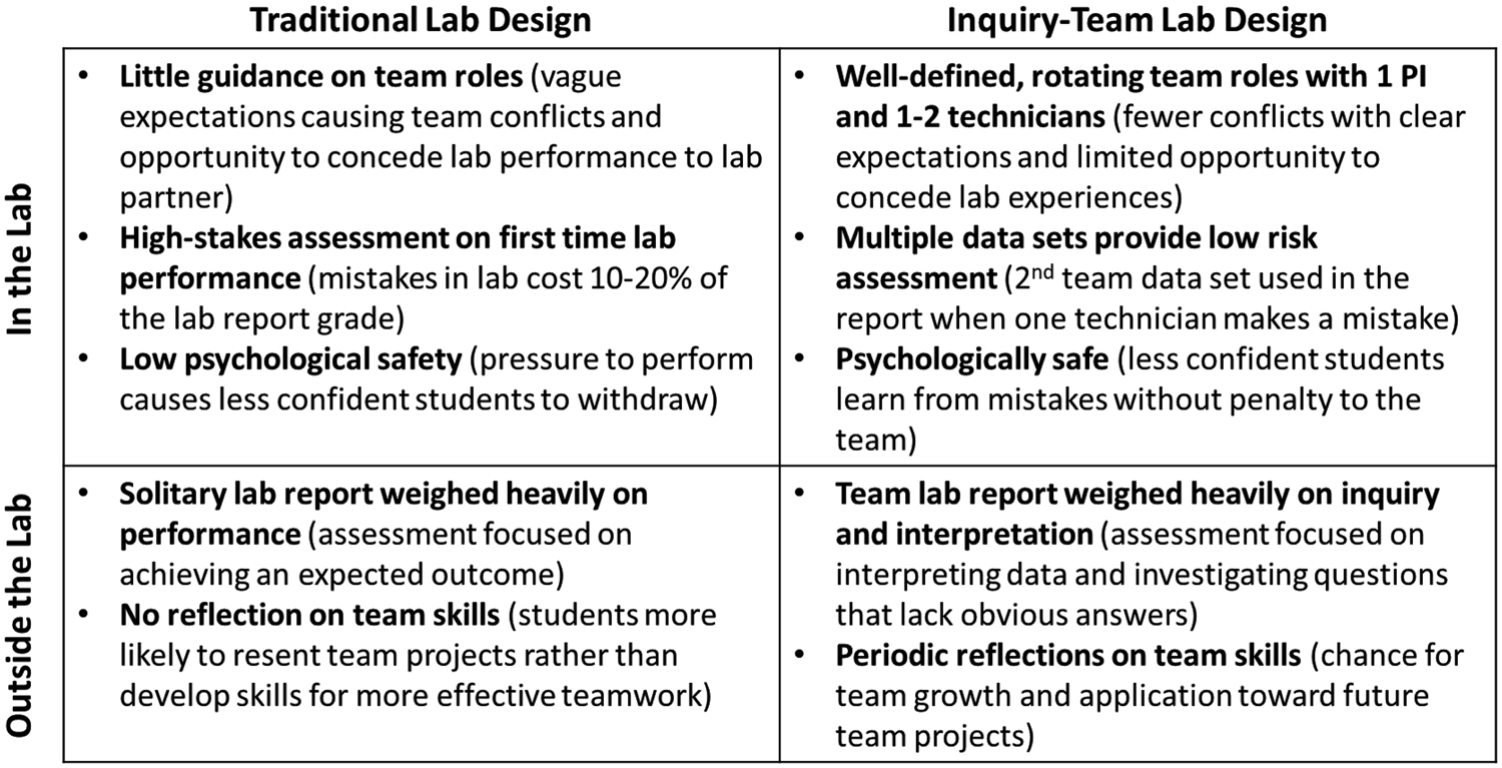
Contrasting properties of the traditionally designed and inquiry-team-based-learning laboratory courses studied

The research team hypothesizes that implementing a collaborative inquiry-team-based learning (ITBL) STEM laboratory course design promotes medical scientist values and behaviors as measured by the experimentally validated Persistence in the Sciences (PITS) survey [1]. Based on learning theories of social constructivism and behaviorism, inquiry-team-based learning seeks to promote growth mindset by providing authentic opportunities to develop students’ scientist identity, the research team aims to close opportunity gaps for systemically minoritized students that did not have access to similar opportunities in their pre-college education. Under ITBL design, student research teams of 2–3 undergraduates are formed with individual roles clearly defined either as laboratory technicians that work individually on experiments or as the principal investigator (PI) responsible for the team’s post-lab write up while providing strictly verbal assistance to technicians that emphasizes the importance of verbal and written communication in science (Fig. 1). During the first week of the course, students are tasked with connecting with teammates to complete a research team agreement that defines the expectations of each role, creates a schedule for rotating the PI role evenly among teammates, determines how and when students will discuss lab data with their PI, and sets expectations for team professionalism including conflict resolution. The opportunity for technicians to develop self-mastery in laboratory skills and provide meaningful contribution to a research project through inquiry-based learning is designed to affirm students’ scientific identity without the previously observed identity-threat-driven interference to concede lab experiments to a lab partner [5, 6, 10]. The technicians then report their individual lab results to the student PI during research team meetings and collaborate on the combined data analysis for the lab reports as part of team-based learning. This duplication of data sets allows technicians the psychological safety to learn from mistakes without jeopardizing the team lab report yet leverage the social motivation to provide high-quality data for the team [5, 11]. Each student also provides a monthly teamwork assessment for each teammate to address team conflicts earlier into the semester and develop conflict resolution skills. In addition to keeping teammates accountable for following the agreed team roles, students build their sense of belonging in the pharmaceutical science community through explicit reflection on their role in the research team, building communication skills, and elucidating facets of hidden curriculum in STEM lab courses through collaboration with a diverse research team [8, 12]. This inquiry-team-based design also authentically reflects professional pharmacy research dynamics where researchers often experiment solidarity, then collaborate on data interpretation as a research team.

## Methods

During three semesters (Spring 2021, Autumn 2021, Spring 2022), the research team recruited junior (rank 3, > 60 credit hours) and senior (rank 4, > 90 credit hours) undergraduates (3+ years into their bachelor’s degree program) enrolled in an ITBL-based pharmaceutical science lab course (*n* = 129) and a traditionally designed biology lab course (*n* = 195) (Table 1). The research team made the conscious decision to compare two separate laboratory courses that ran simultaneously during the study period to control for historic threats to validity from offering one lab design more proximal to the start of the COVID-19 pandemic and its observed learning interruptions, as well as eliminate potential resentful demoralization from students in differently designed sections within the same pharmaceutical sciences lab course. In both lab courses, STEM major students attended one, 3-h lab section each week in addition to 3 h of lecture and both courses required previous or simultaneous enrollment in chemistry courses (general chemistry for the traditional lab, organic chemistry for the ITBL lab). In the traditional lab course, students worked in groups of 3–5 to complete an inquiry-based lab exercise, answer questions, and submit one lab report at the end of the lab period. Students did not have explicit rotating roles during the lab, instead they worked together to complete one data set for any given experiment. The two lab courses otherwise had comparable course structure of lectures, laboratory activities, lab reports, and examinations. Exclusion criteria included a lack of student consent to be included in the study or incomplete sections on the Persistence in the Sciences (PITS) survey. This study was deemed IRB-exempt by the Ohio State University’s Office of Responsible Research Practices.

**Table 1:** Demographic information for consenting students by course (total *n* = 340; traditional *n* = 202, ITBL *n* = 138)

**Variable**	**Traditional** ***n*** **(%)**	**ITBL** ***n*** **(%)**	***X***^2^ **test** ***p*** **value**
Sex			0.043
Female	125 (61.9)	100 (72.5)	
Male	77 (38.1)	38 (27.5)	
Rank			< .001
3	134 (66.3)	16 (11.6)	
4	68 (33.7)	115 (83.3)	
Missing	0 (0)	7 (5.1)	
First generation status			0.487
First generation	45 (22.3)	35(25.4)	
Continuing generation	157 (77.7)	102 (73.9)	
Missing	0 (0)	1 (.7)	
Ethnicity			0.096
Asian	15 (7.4)	23 (16.7)	
Black or African American	15 (7.4)	13 (9.4)	
Hispanic	9 (4.5)	4 (2.9)	
Non-resident alien	8 (4.0)	8 (5.8)	
None given	5 (2.5)	5 (3.6)	
Two or more races	8 (4.0)	7 (5.1)	
White	142 (70.3)	78 (56.5)	

	Traditional Mean (SE)	ITBL Mean (SE)	MANCOVA *p* value
Final course grade	3.25 (.0591)	3.62 (.0719)	< .001
Final chemistry grade	2.71 (.1059)	2.76 (.0951)	0.732
BOT GPA	3.36 (.0618)	3.39 (.0411)	0.726

*BOT* beginning of term, *GPA* grade point average

Sex, ethnicity, first generation status, GPA, chemistry grades, and rank information were obtained from university databases. Due to small sample size among some groups enrolled in a predominantly white institution, multiple semesters of course offerings were collected and students were categorized into white or non-white ethnicity (Table 1). Students reporting as non-resident aliens, none given, or two or more races were assigned to the non-white category. While this is making broad assumptions about student experiences in the non-white group, students in the non-white category do face varying degrees of identity threat at a predominantly white institution such as the study site.

At the end of either STEM lab course, students completed the experimentally validated, 40 question PITS survey, which was designed to determine student perceptions in six themes that are predictive of persistence in the sciences [13]. The six sections on the PITS include Project Ownership-Content, Project Ownership-Emotion, Science Self-Efficacy, Science Identity, Scientific Community Values, and Networking. Based on the persistence framework, these six sections of the PITS reflect factors for students that lead to staying in STEM [13, 14]. In the persistence framework, research experiences, active learning, and community-based learning contribute to the learning of science and formation of scientific identity [14]. While previous studies attributed student motivation and confidence to persistence in STEM, these six predictors of persistence also reflect values and behaviors which are hypothesized to attribute to professional identity formation (Fig. 1) [13, 15, 16]. The PITS survey was designed to be a tool to evaluate these student outcomes as a result of participation in educational programs such as research experiences [13]. Students were given a small amount of course bonus points to complete the survey (< 1% of the final grade).

In this quasi-experimental design, quantitative univariate and multivariate analyses were used to examine differences between ITBL and traditional lab students, controlling for academic factors (introductory chemistry grades, beginning of term GPA), and demographics (first generation status, white/non-white, gender, and rank). Due to low sample size among certain groups, *t*-tests were used to make comparisons between groups among specific subsets such as first-generation students, ethnicities, and gender. Assumptions of statistical tests were met unless otherwise noted. The Bonferroni correction for multiple tests was used where appropriate.

In Autumn 2021 and Spring 2022, the research team also included an open-ended response question on the survey to determine what factors may have contributed to student PITS scores. Students in both groups were asked the following prompt: “Please describe an event that helped you to realize ‘I am a scientist’ or if none comes to mind, was there a time that made you think ‘I might not be a scientist after all. Did this happen during a STEM course or outside a STEM course?’”.

Qualitative analysis of the open-ended question was conducted by developing a codebook of emergent common themes. One researcher (AK) developed the codebook using the Autumn 2021 responses, and the second (ND) used that codebook to evaluate all Autumn 2021 student responses. An interrater reliability analysis using Cohen’s kappa determined consistency among raters. All code mismatches were discussed to consensus, and the codebook was revised to match the discussion, including adding and condensing themes. Using this revised codebook, both raters coded the Spring 2022 data separately, and the interrater reliability was calculated for this portion of the data, and again, all mismatches were discussed to consensus. Combining the two semesters, and using these themes, the traditional and ITBL students were compared regarding student scientific identity and their explanations behind feeling like a scientist or not, and where the events that supported (or hindered) this feeling took place (e.g., in a STEM course, outside a STEM course, or both). Demographic comparisons were also evaluated within each group.

## Results

A total of 202 traditional and 138 ITBL students consented to participate in the study. Student sex was slightly more female in the ITBL course (Table 1). First generation status was similar among the two groups, but rank differed significantly, with more rank 4 students in ITBL. Ethnic identities among the students in both courses were mostly white, and although the ITBL course appears more diverse, this was not significantly different. Students in both groups had similar prior academic performance based on beginning of term (BOT) grade point averages (GPA) and first college chemistry grades, though ITBL students earned higher final course grades on average than the traditional lab students.

**Fig. 2:**
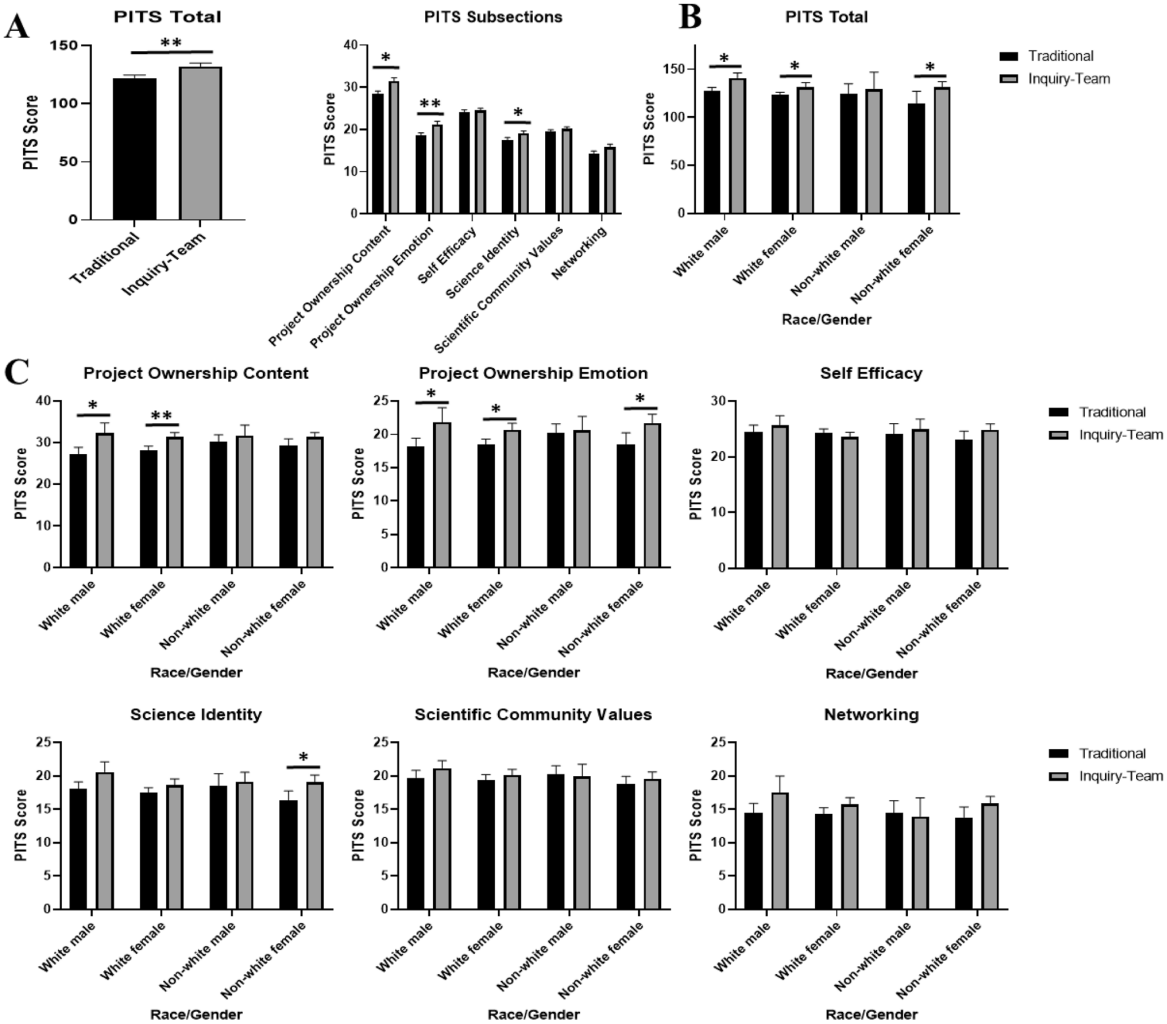
Persistence in the Sciences (PITS) total and subsection scores between traditional vs ITBL designed labs (**A**) and by race/gender total PITS (**B**) and PITS subsections (**C**). *0.001 < *p* < 0.008; **p < 0.001

Total and individual PITS section scores were compared between the two courses, controlling for academic performance and demographics. A total of 136 traditional and 77 ITBL students had complete data for this analysis. ITBL-design lab reported significantly higher overall PITS scores (ANCOVA, Fig. 2). Of the six sections of the PITS, significantly higher scores were observed for the ITBL students in Project Ownership-Content, Project Ownership-Emotion, and Science Identity.

**Fig. 3:**
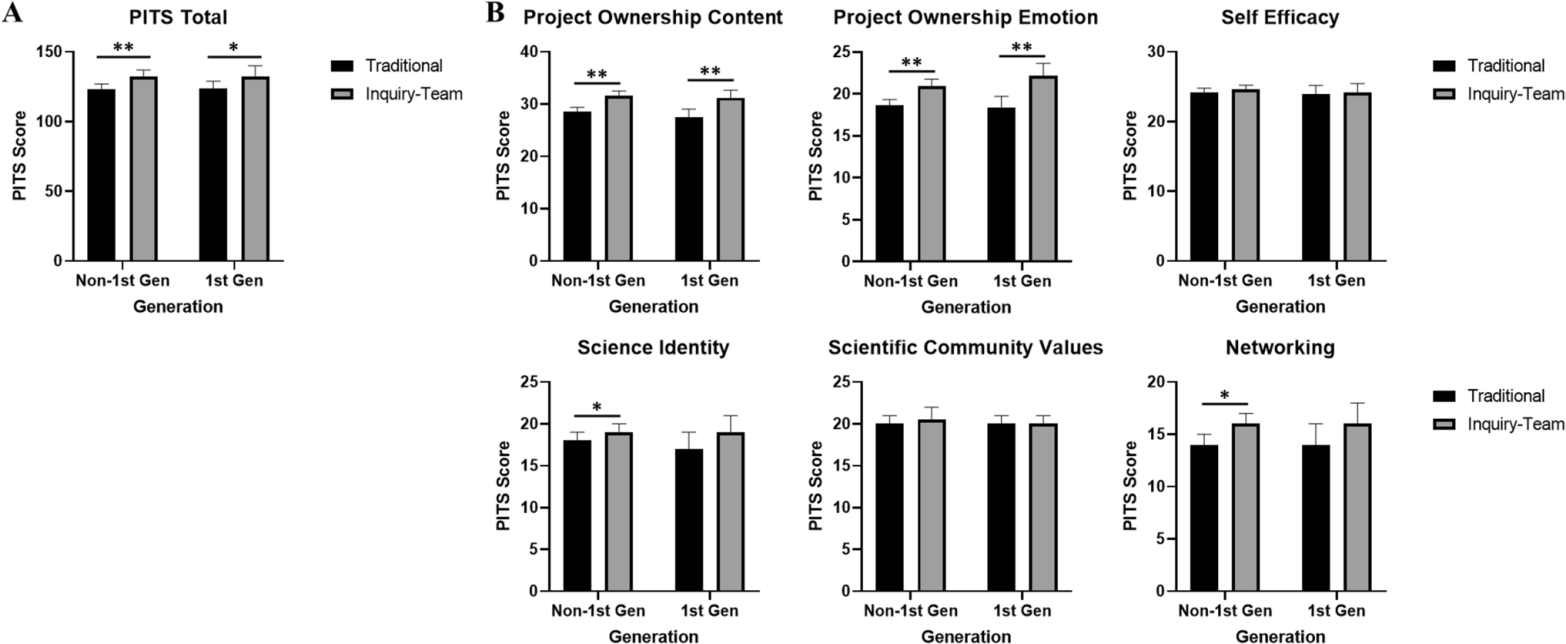
PITS score: total (**A**) and subsection (**B**) scores between traditional and ITBL designed labs by first-generation college student status. Non-1st Gen, continuous generation. *0.001 < *p* < 0.008; **p < 0.001

The *t*-tests indicate significantly higher overall PITS scores in ITBL white males (+10.0%), white females (4.8%), non-white female (+7.6%), and first-generation students (+5.9%) compared to traditional lab design (Fig. 2). For white females, the largest PITS scores were in the Project Ownership-Content (+7.7%) and Project Ownership-Emotion (+7.5%) categories for students in ITBL over traditional labs. ITBL non-white females reported higher scores in the Science Identity (+11%) and Project Ownership-Emotion (+11.6%) categories. For white male students, Project Ownership-Content indicated some of the largest differences between ITBL and traditional labs (+12.5% points). ITBL first-generation students had the greatest enhancement in the Project Ownership-Content (+9.2%) and the Project Ownership-Emotion categories (+12.7%, Fig. 2). While non-white males in ITBL labs demonstrated positive trends in PITS categories, they did not achieve statistically significant differences. ITBL continuing generation students also had significantly higher PITS scores than traditional students in the Project Ownership-Content (+7.6%), Project Ownership-Emotion (+7.6%), Science Identity (+6.2%), and Networking (+7.0%) categories (Fig. 3).

For the four parts of the open-ended student responses, Cohen’s kappa for the Autumn 2021 data ranged from .142 to .767. Low agreement (kappa = .142) was observed for Code 2 because there were disagreements about the existence of a second code. For Code 1 (.651), STEM (.574), and Scientist (.767), kappa indicates moderate to substantial agreement among the two raters.^10^ The interrater reliability for the Spring 2022 responses indicated greater consensus with the second round of coding (Code 1 = .591, Code 2 = .547, STEM = .713, Scientist = .846).

For the open-ended question, 155 traditional students and 84 ITBL students provided responses. Students were categorized into *Scientist*, *Not a scientist*, *Both* (a scientist and not a scientist), and *Unknown*. A chi-square test indicates these groups are different among the two lab types (*p* = .015). ITBL students had a higher incidence in science identity (76.2% vs 65.2%) because they *Gained confidence* (30.9% vs 18.7%; e.g., understood the science behind a lab experiment), saw S*cience as a way to contribute to society* (13.1% vs 3.9%; e.g., saw the experiments applying to real-world problems in pharmacy), and very few ITBL students reported that they *Disliked an aspect of the scientific process* (2.3% vs 5.16%; e.g., disliked data analysis). For traditional lab students who identified as scientists, most responses were coded into one of four categories: *Gained confidence*, *Liked an aspect of the scientific process* (e.g., completing class related labs), had a *Positive external experience* (e.g., working in a research lab), and they felt *Science is joyful/interesting* (e.g., having a passion for science). Similarly, ITBL students identifying as *Scientists* most often identified the following reasons: *Gained confidence*, *Liked an aspect of the scientific process*, and had a *Positive external experience.* However, ITBL students more often referred to *Science as a way to contribute to society* (e.g., contributing to solving world problems) as contributing to their development of a science identity (Table 2). For students identifying as a *Scientist*, the majority (67.3% of traditional, 65.6% of ITBL) stated that the events supporting this took place inside a STEM classroom.

**Table 2:** Comparison of the number (% total) of student responses per parent code for traditional and ITBL students

	**Number (% of total) of student responses**		
**Parent code**	**Traditional** **(*n* = 155)**	**ITBL** **(*n* = 84)**	***X***^**2**^ ***p*** **value**
Dislike an aspect of the scientific process	8 (5.2)	2 (2.4)	.305
Experienced failure	7 (4.5)	5 (6.0)	.627
Need to do “X” to be a scientist	13 (8.4)	5 (6.0)	.496
Negative external experience	2 (1.3)	2 (2.4)	.530
Self-doubt	8 (5.2)	4 (4.8)	.893
Positive external experience	19 (12.3)	14 (16.7)	.346
Science as a way to contribute to society	6 (3.9)	11 (13.1)	.008
Science is joyful/interesting	20 (12.9)	9 (10.7)	.528
Good at science	6 (3.9)	5 (6.0)	.463
Gained confidence	28 (18.1)	26 (31.0)	.032
Like an aspect of the scientific process	67 (43.2)	41 (48.8)	.355

“X”, haven’t contributed to knowledge to the scientific community; learning the basics (know so little); no research yet (what they view as “real” research); not enough experience

More students in the traditional lab (20.6% of traditional, 5.9% of ITBL) identified as *Not a Scientist*. Traditional lab students mentioned they had *Experienced failure* (e.g., experiment went “wrong”, they earned poor grades), they *Disliked an aspect of the scientific process*, or they *Need to do “X” to be a scientist* (e.g., they have not conducted “real” research yet) as reasons they were *Not a scientist*. While ITBL also cited the *Need to do “X” to be a scientist*, they differed from traditional lab students by providing examples of *Negative external experiences* (e.g., a bad internship experience) and having *Self-doubt* (e.g., feeling incompetent). The events contributing to students feeling like *Not a Scientist* most often took place (59.3% of traditional, 100% of ITBL) in the STEM classroom.

Several students in both groups identified as *Both* (5.8% of traditional, 10.7% of ITBL) a scientist and not a scientist. Traditional lab students most often cited having *Experienced failure* as the reason they are *Not a scientist*, and that *Science is joyful/interesting* for identifying as a *Scientist*. ITBL students in the *Both* category most frequently cited *Self-doubt* as the reason for identifying as *Not a scientist* and *Gaining confidence* for identifying as a *Scientist*. When the researchers could not discern the students’ responses or reasoning, they were assigned to the *Unknown* category (8.4% of traditional, 7.1% of ITBL).

In the traditional lab, 61.3% of non-white students identified as a *Scientist*, compared with 65.7% of white students. In ITBL, 62.5% of non-white students identified as a *Scientist*, compared with 84.6% of white students. In the traditional labs, non-white students explained they felt like a *Scientist* because they *Liked an aspect of the scientific process* and *Gained confidence*, which were also the top two reasons given by the non-white ITBL students who identified as a *Scientist*.

## Discussion

Inquiry-team-based learning remains underutilized in health professional education despite the rising interest in active learning, interprofessional competencies, and professional identity formation. While problem-based learning such as case studies and reflections on healthcare practice predominates healthcare professional programs, inquiry-based student-led research investigations improve student learning through authentic discovery in addition to promoting medical scientist identity by socializing students to pharmaceutical science investigation [15–18]. Team-based learning’s mix of individual contributions and team reporting develops the communication skills assessed on team-readiness assurance tests (tRATs) that are widely recognized as vital for medical scientists interacting with an interdisciplinary healthcare team [19]. However, inquiry-based team experiences that give students a sense of community in pharmaceutical innovation are rarely utilized.

This study’s results support the hypothesis that inquiry-team-based learning designed lab courses enhance student persistence in the science (PITS) through an authentic research team experience (Fig. 1). While the ITBL designed pharmaceutical sciences lab average final grade was higher than the traditionally designed biology lab, the research team is hesitant to claim ITBL lab design enhances student performance, instead, it may be due to the differences in ITBL and traditional course content. However, students of all racial, gender, and college generation demographics in the ITBL labs had significantly higher PITS scores with exception of non-white male students, which had the smallest enrollment that limited the study’s statistical power. The trends in PITS scores observed in this study are similar to others comparing research-based practices such as Course-based Undergraduate Research Experiences (CUREs) or field experiences with traditional courses [20, 21]. This challenge in non-white male recruitment reflects the national challenge of recruiting and retaining underrepresented male students in undergraduate STEM programs during the 2020 syndemic of COVID-19 and increased racial tensions [22].

ITBL lab design provided both universal and unique benefits to aspects of persistence in the sciences among the student demographic groups. With exception of non-white males, ITBL lab students demonstrated significantly higher subsection PITS scores in Project Ownership-Emotion while white students had higher Project Ownership-Content and non-white female students reported higher Science Identity. Interestingly, both first-generation and continuous generation students demonstrated enhanced Project Ownership-Content and Project Ownership-Emotion, but only continuous generation students demonstrated enhanced Science Identity and Networking in the ITBL lab. The qualitative survey of students’ identify-affirming experiences suggests that the enhanced metrics of persistence in the sciences are likely due to the ITBL design providing more opportunity for students to develop an appreciation for science’s contributions to society and gaining confidence as a scientist.

Previous studies demonstrate that a growth mindset learning environment provides the psychological safety for students to learn the scientific process by shifting assessment away from first-time technical performance in the lab and instead toward authentic inquiry that includes data interpretation and protocol optimization [5, 23, 24]. This realignment toward more authentic inquiry for pharmaceutical scientists likely gave non-white female and continuous generation students more opportunity to build their science identity by minimizing the impact of identity threat through explicit team roles, resulting in more personal achievement and confidence in lab [14–16]. The balance between psychological safety and teammate accountability from the explicit team roles likely gave all students a sense of ownership in their learning and progress from social motivation to perform well in lab into personal intrinsic motivation for continuous learning and skill development [5, 25]. Similarly, the authentic research investigations with emphasized importance to society’s pharmaceutical use and focus on science communications both between teammates and in scientific writing may have contributed to a higher sense of networking in continuous generation ITBL students that parallels advocacy for medical science.

Surprisingly, this ITBL lab offering did not observe higher student scores on the Self-Efficacy, Scientific Community Values, nor Networking that other inquiry-based learning pedagogies have demonstrated [20, 21, 26]. This could be in part due to pharmaceutical science labs being offered to rank 3 or higher undergraduates, who likely already gained these facets of persistence or dropped from their STEM major as a part of survivorship bias [9, 21]. Similarly, non-white student Project Ownership Content and first-generation Science Identity and Networking scores did not increase compared to their majority counterparts; therefore, further course reiteration is required to explicitly promote these predictors of persistence in the sciences to overcome challenges to non-white and first-generation persistence in the sciences.

While the two lab designs share similar lecture and lab frequencies and were both reported as pivotal experiences for all STEM students, it remains unclear whether a biology or pharmaceutical science content-focused course attracts more students with a stronger sense of science identity or project ownership. While the ITBL labs recruited more rank 4 equivalent and female students to this study, the racial demographics were comparable between the two study groups with a slight trend toward more Asian students recruited in the ITBL lab. It is unclear whether students with a longer tenure at the university had more opportunities to experience a positive outside influence on their science identity or whether female and Asian students develop a net stronger science identity as part of racial/gender groups that are overrepresented in pharmaceuticals sciences but underrepresented in pharmacy leadership [27, 28]. Although we compared students of similar rank, learners who choose a pharmacy lab course might be at a different place of professional identity formation than learners in a general biology lab course, which may be worth exploring in future studies.

In addition, the COVID-19 pandemic was particularly strenuous on healthcare staff who were mobilized into assisting the national vaccination efforts with sources of burnout including longer uncompensated work hours, isolation from support networks, and fear of catching/spreading COVID-19 [27, 29]. However, pharmacists also reported an increased sense of professional identity due to the professional achievement and recognition of pharmacists as healthcare professionals during the pandemic [2, 27]. It remains unclear whether the net impact of these outside influences externally socialized pre-pharmacy undergraduate students to pharmacists’ societal importance that then enhanced students’ medical scientist identity [15, 25].

## Conclusion

While many examples of laboratory instruction include components of inquiry-based and team-based learning, this study is one of the first to demonstrate the effect of explicit inquiry-team-based laboratory design on measures of student persistence in the pharmaceutical sciences and their connection to scientist identity formation. The pharmaceutical sciences ITBL lab offered a collaborative, growth-promoting environment with experiments that were inquisitive and impactful to perspective pharmacists and pharmaceutical scientists, which resulted in students reporting higher levels of predictors to persistence in the sciences such as Project Ownership-Content, Project Ownership-Emotion, Science Identity, and Networking across various student demographics. This suggests that ITBL labs emulating professional research experiences may also prepare students with the investigation and teamwork skills to pursue undergraduate research and professional internships that further enhance students’ medical scientist identity.

While there are limitations due to outside influences of the combined COVID-19 pandemic and the resurgence of racial tensions in the USA during the study, the impact of ITBL lab design has persevered in such challenging environments. In addition to eliciting future research on the impact of ITBL lab design on long-term student persistence in STEM-related careers, it would also be interesting to investigate the impact of ITBL design in other fields and through other derivations of ITBL-lab design such as CUREs and process oriented guided inquiry learning (POGIL) on developing STEM persistence indicators that overlap with professional identity formation.

## Data Availability

The corresponding author can provide the datasets used in this study at reasonable request.
